# Both donor and recipient sex determine behavioral and neuroimmune outcomes in a gut microbiota transfer experiment from the unpredictable chronic mild stress model of depression

**DOI:** 10.1016/j.bbih.2026.101248

**Published:** 2026-04-27

**Authors:** Meagan E. Hinks, Alexandre S. Maekawa, Mark D. Corrigan, S.M. Nageeb Hasan, Derek Wan-Yan-Chan, Tanya Nadine Burry, Stephanie Salia, Francine F. Burke, Francis R. Bambico, Ashlyn Swift-Gallant

**Affiliations:** Faculty of Science, Memorial University of Newfoundland, 232 Elizabeth Ave., St. John's, Newfoundland and Labrador, A1B 3X9, Canada

**Keywords:** Gut–brain axis, Sex differences, Gut microbiota, Chronic stress, Depression, Neuroimmune signaling

## Abstract

Sex differences in major depressive disorder (MDD) are well established, yet the conditions under which immune dysfunction contributes to depression—and how this differs by sex—remain poorly defined. The gut–brain axis represents a key immune-mediated pathway linking chronic stress to depressive phenotypes, but sex is rarely considered at the level of the microbial source and host susceptibility. Here, we assessed whether transfer of stress-altered gut microbiota is sufficient to induce alterations in depression-relevant outcomes by using a cross-sex a cecal microbiota transfer (CMT) from mice exposed to unpredictable chronic mild stress (UCMS). Female recipients exhibited greater anhedonia- and despair-like behaviors following UCMS CMT, indicating a sex-specific risk to immune–microbial perturbation. Donor sex further modulated these effects: microbiota derived from stressed females preferentially transmitted a depressive-like phenotype, whereas stressed male microbiota was associated with attenuated neuroinflammatory profiles with no changes in behavior. In contrast, male recipients showed limited behavioral change but displayed pronounced alterations in neuroimmune and prefrontal monoamine gene expression with female UCMS microbiota. Together, these findings demonstrate that immune-mediated depressive phenotypes emerge under sex-dependent conditions defined by both donor and host biology. Furthermore, females may be particularly responsive to gut-targeted immunomodulatory interventions, while males exhibit immune and monoaminergic transcriptional sensitivity without overt behavioural expression. By identifying sex-specific immune and microbiota-associated mechanisms, this work informs the design of future immunotherapy trials for depression, highlighting the gut as a tractable and potentially sex-selective therapeutic target.

## Highlights

• Cross-sex microbiota transfer was performed using the UCMS model of depression
• Stress-altered microbiota induced depressive-like behavior selectively in females
• Males treated with female UCMS cecum displayed IL-2/IL-17a immune activation
• Males showed selective alteration in neural monoaminergic gene receptor expression
• Findings support sex-specific and host-recipient effects in gut microbiota transfer experiments

## 1 Introduction

Major depressive disorder (MDD) is a leading cause of global disability (GBD Mental Disorders Collaborators, 2022; Desai Boström et al., 2025). Although women experience MDD at nearly twice the rate of men, foundational preclinical research informing current therapies has predominantly relied on male models (GBD, 2019 Mental Disorders Global Burden of Disease 2019 Mental Disorders Collaborators, 2022; Li et al., 2023). This imbalance may contribute to limited treatment efficacy and reproducibility, particularly for those most affected.

Emerging evidence implicates gut microbiota as a mechanistic contributor to MDD via the gut–brain axis (GBA; Carabotti et al., 2015; Donoso et al., 2023). Clinical and preclinical studies consistently report a pro-inflammatory gut profile in MDD, mirroring microbial shifts observed in stress-exposed rodents (Aizawa et al., 2016; Chen et al., 2018; Jiang et al., 2015, 2025; Marin et al., 2017; Zheng et al., 2016). Enteric inflammation influences central neuroimmune signaling through cytokine production, gut-derived metabolites, and impaired intestinal barrier integrity, contributing to systemic inflammatory dysregulation and neuroinflammation (Maes et al., 2008; Okumura and Takeda, 2017; Xu et al., 2022).

Gut microbiota additionally regulates the availability of amino acid precursors required for monoaminergic neurotransmitter synthesis, including serotonin, dopamine, and norepinephrine—key targets of antidepressant therapies (Hardebo and Owman, 1980). Inflammatory microbial states disrupt neurotransmitter synthesis precursors including tryptophan and phenylalanine-derived tyrosine availability, effects that are reversible through probiotic interventions that restore gut integrity and reduce inflammation (Chen et al., 2021; Marin et al., 2017; Wu et al., 2022; Zhou et al., 2023).

Causal evidence for gut involvement in MDD is provided by fecal microbiota transfer (FMT) studies, in which microbiota from MDD patients or stressed animals induce depressive-like behaviors and neuroinflammatory signaling in recipients, whereas transfer from healthy donors partially reverses stress-induced phenotypes (He et al., 2024; Chevalier et al., 2020; Li et al., 2019; Marin et al., 2017; Zhou et al., 2023). Despite well-documented sex differences in both MDD prevalence and gut microbiota composition, most FMT studies rely on same-sex transfers, typically male-to-male, and do not account for sex as a biological variable (Li et al., 2019; Zhou et al., 2023). Beyond our prior work demonstrating sex-dependent behavioral effects of cross-sex microbiota transfer (Hinks et al., 2023), only one study has examined cross-sex transfer, focusing on metabolic rather than behavioral outcomes (Feješ et al., 2024).

Here, we test whether donor or recipient sex modifies the behavioral, neuroimmune, and monoaminergic measures in a cross-sex gut microbiota transfer depression model. First, cecum was collected from donor mice exposed to six weeks of unpredictable chronic mild stress (UCMS). Next, recipient mice received either saline (control) or cecum from either male or female UCMS mice via oral gavage. Cecum recipient mice then underwent behavioral testing for depressive- and anxiety-like phenotypes, followed by assessment of neuroimmune cytokine signaling and immune- and monoaminergic-related gene expression in the prefrontal cortex, a region central to stress and depression pathophysiology (Pizzagalli and Roberts, 2022). We hypothesized that transfer of microbiota from UCMS donors would alter behavioral phenotypes in a donor sex-dependent manner: female-derived UCMS microbiota would exacerbate MDD-like behaviors, including reduced pleasure-seeking and grooming, whereas male-derived UCMS microbiota would increase behavioral despair (Hinks et al., 2023). Finally, we hypothesized that neuroimmune and gene expression changes would parallel behavioral outcomes, revealing pathways through which gut microbiota confer sex-specific vulnerability to chronic stress. By identifying sex-specific microbial mechanisms associated with vulnerability to chronic stress, this study addresses a critical gap in understanding the role of the microbiome in preclinical models of depression.

## 2 Materials and methods

### 2.1 Animals

Male and female BALB/c mice were obtained from Charles River Laboratories (QC, Canada) and housed under controlled temperature and humidity on a 12 h light/dark cycle with ad libitum access to food and water. Mice were singly housed upon arrival to the facility to minimize cage-effects on microbiome transfer via coprophagy (Russell et al., 2022). All procedures were approved by the Institutional Animal Care Committee at Memorial University of Newfoundland in accordance with Canadian Council on Animal Care guidelines. Full methodological details can be found in the Supplementary Methods.

### 2.2 Cecal donors and unpredictable chronic mild stress

**Fig. 1:**
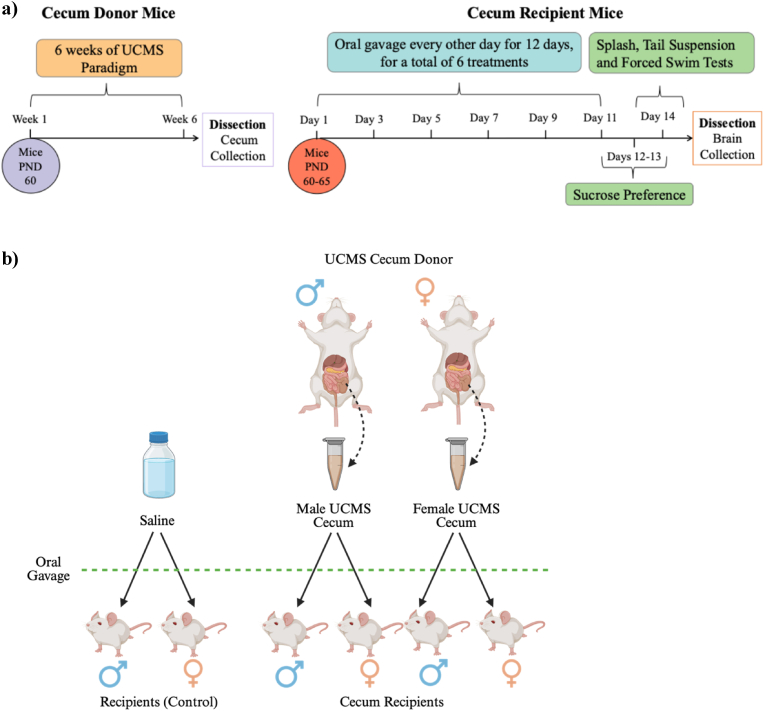
Schematic of **a)** experimental timeline and **b)** treatment groupings.

Cecal donor mice (n = 6–8 per sex) were exposed to a six-week unpredictable chronic mild stress (UCMS) paradigm beginning at postnatal day (PND) 60, consisting of varied physical, psychological, and circadian stressors (Fig. 1a). Cecal contents were collected one day following UCMS completion, pooled by donor sex, diluted in phosphate-buffered saline, and stored at −80 °C until transfer.

### 2.3 Cecal microbiota transfer

Sixty recipient mice received oral gavage of sex-pooled cecal microbiota derived from male or female donors every other day for 12 days, with saline administered as a control (six treatments in total; Fig. 1a). Treatment groups included male-derived microbiota, female-derived microbiota, or saline, with equal numbers of male and female recipients per group (n = 10/sex/group; Fig. 1b). Behavioral testing commenced 24 h after the final gavage, followed by tissue collection.

### 2.4 Behavioral assessment

Cecal recipient mice underwent a battery of validated assays assessing depressive- and anxiety-like behaviors, including the sucrose preference test, splash test, tail suspension test, and forced swim test, administered across three consecutive days. Behavioral scoring was performed using a combination of manual coding and automated tracking software.

### 2.5 Neuroimmune and gene expression analyses

Following behavioral testing, prefrontal cortex (PFC) tissue was collected for assessment of neuroimmune signaling and gene expression. Pro-inflammatory cytokines and chemokines were quantified using multiplex immunoassays, and expression of immune- and monoaminergic-related genes was measured via quantitative PCR.

### 2.6 Statistical analysis

All statistical analyses were performed using Jamovi (Version 2.6.45; The Jamovi project, 2025). Behavioural measures were assessed using between-subjects analysis of variance (ANOVA) to compare groups based on sex, treatment, and sex by treatment interactions. For analyses of chemo-cytokine and gene expression, a Principal Component Analysis (PCA) using Oblimin rotation parallel analysis was performed on each measure to determine factor component groupings. For gene expression analyses, delta CT values were calculated and normalized to the housekeeping gene, GAPDH. Component scores were then evaluated with ANOVA to evaluate sex and/or treatment effects. Significant effects were followed up by Fishers Least Significant Difference (LSD), with alpha set at *p* < .05. Post hoc p-values were adjusted for multiple comparisons using the Benjamini-Hochberg procedure to control the false discovery rate at 15% (q < 0.15).

## 3 Results

### 3.1 Anhedonia in response to microbiota from UCMS females: up in males, down in females

**Fig. 2:**
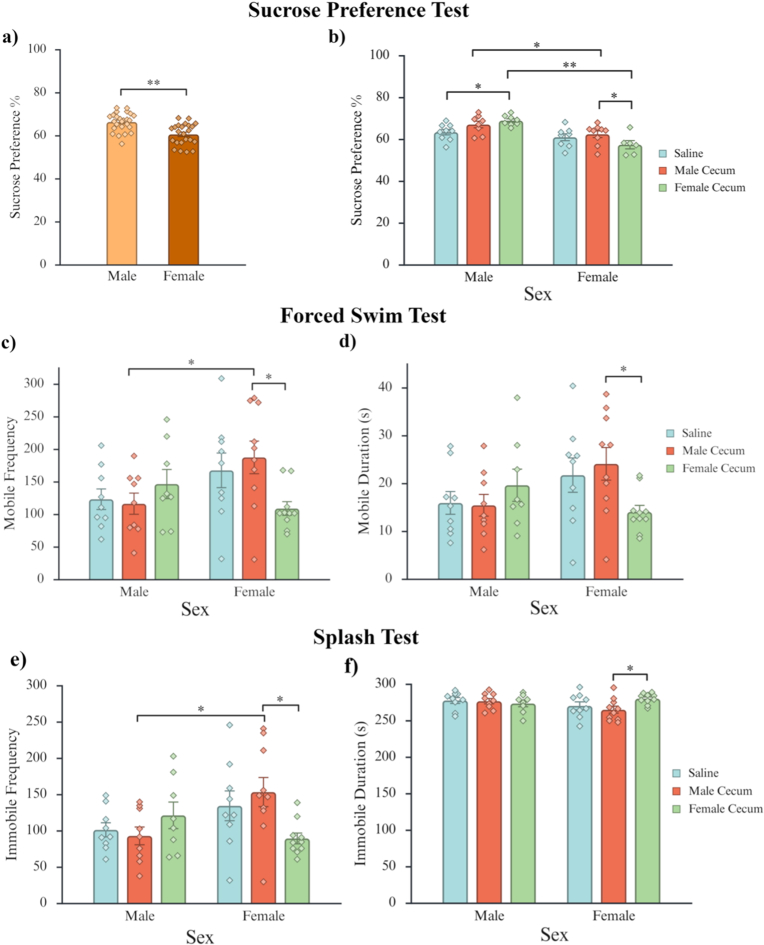
Behavioural data for the sucrose preference and forced swim tests. Note. Individual data points for each observation are shown along with the mean ± standard error of the mean (SEM). **a)** Male mice had significantly higher sucrose preference scores compared to females **b)** Females treated with either female cecum or male cecum had lower sucrose preference scores than males of the same treatment. Females treated with female cecum had lower preference scores compared to females treated with male cecum. Males treated with female cecum had higher preference scores compared to male controls. **c)** Female mice treated with female cecum had significantly lower mobile frequencies compared to females treated with male cecum. Females treated with male cecum had significantly higher frequencies of mobility than males of the same treatment. **d)** Females treated with female cecum had significantly lower mobile durations than females treated with male cecum. **e)** Female mice treated with female cecum had significantly lower mobile frequencies compared to females treated with male cecum. Females treated with male cecum had significantly higher frequencies of immobility than males of the same treatment. **f)** Female mice treated with male cecum had significantly lower immobile durations compared to females treated with female cecum. *∗ = p < .05, ∗∗ = p < .001*.

For sucrose preference scores on the SPT, a significant main effect of sex was found F (1,42) = 24.955, p < .001, η^2^ = 0.315, where male mice had significantly higher sucrose preference scores than females (Fig. 2a). There was also a significant interaction between sex and treatment on preference scores, F (2,42) = 4.588, *p* = .016, η^2^ = 0.116 (Fig. 2b). Post-hoc analysis on the interaction of treatment and sex showed that females treated with female UCMS cecum had the lowest scores, significantly lower than both males with the same treatment, *p* < .001, FDR adjusted *p* = .010, Cohen's d = 2.707, 95% CI [−3.949, −1.465], and females treated with male UCMS cecum, *p* = .031, FDR adjusted *p* = .103, Cohen's d = 1.180, 95% CI [−2.274, −0.085]. Additionally, males treated with female UCMS cecum had higher sucrose preference scores than male controls *p* = .011, FDR adjusted *p* = .037, Cohen's d = 1.295, 95% CI [0.274, 2.316]. Male cecum was also found to have significant effects dependent on recipient sex, with females treated with male UCMS cecum having lower preference scores compared to males with the same treatment, *p* = .030, FDR adjusted *p* = .100, Cohen's d = 1.090, 95% CI [2.099, 0.080].

### 3.2 Sex-dependent behavioral effects on the forced swim test: mobility up in females, down in males in response to female UCMS microbiota

Analyses of mobility measures on the FST revealed significant interactions between sex and treatment on mobile frequency, *F* (2,49) = 3.913, *p* = .027, η^2^ = 0.129, mobile duration, *F* (2,49) = 3.549, *p = *.036, η^2^ = 0.121, and immobile frequency, *F* (2,49) = 4.635, *p = *.014, η^2^ = 0.149, while the sex and treatment was found for immobile duration was trending, *F* (2,49) = 2.535, *p* = .090, η^2^ = 0.088 (Fig. 2c–f). For mobile frequency, female UCMS cecum significantly decreased frequency in recipient females compared to females treated with male cecum, *p* = .006, FDR adjusted *p* = .079, Cohen's *d* = 1.287, 95% CI [0.351, 2.223] (Fig. 2c). Additionally, females treated with male UCMS cecum had higher frequencies of mobility compared to males of the same treatment, *p* = .014, FDR adjusted *p* = .084, Cohen's d = 1.166, 95% CI [−2.119, −0.213]. For mobile duration, females treated with female UCMS cecum had significantly lower mobile durations compared to females treated with male UCMS cecum, *p = *.011**,** FDR adjusted *p* = .079, Cohen's *d* = 1.180, 95% CI [0.250, 2.111] (Fig. 2d).

Post-hoc analyses between sex and treatment for immobile frequency showed that females treated with female UCMS cecum had lower immobile frequencies compared to females treated with male UCMS cecum, *p* = .004, FDR adjusted *p* = .079, Cohen's *d* = 1.362 95% CI [0.422, 2.303] (Fig. 2e). Male cecum was also found to have significant effects dependent on recipient sex, as females treated with male UCMS cecum had significantly higher frequencies than males of the same treatment, *p* = .007, FDR adjusted *p* = .079, Cohen's *d* = 1.287, 95% CI [−2.247, −0.328]. Post-hoc tests for the interactions for immobile duration showed that females treated with male UCMS cecum had less time immobile than females with female UCMS cecum, *p = *.011**,** FDR adjusted *p* = .079, Cohen's *d* = 1.180, 95% CI [0.250, 2.111], respectively (Fig. 2f). No significant main effects or interactions were found in either the ST or TST, p > .05.

### 3.3 Male UCMS microbiota attenuates neuroinflammation, while female UCMS microbiota Amplifies T-cell–linked inflammation in males

**Fig. 3:**
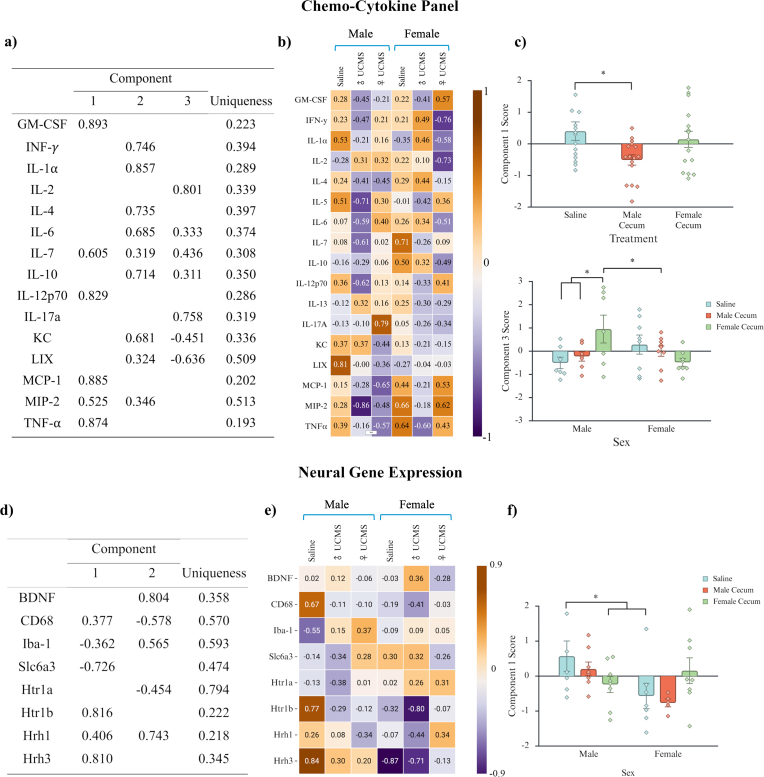
Analysis of chemo-cytokines and neural gene expression within the PFC. Note. Data presented represents component weights determined through PCA using an Oblimin rotation (δ = 0), based on parallel analysis. Tables **a** and **d** represent the overall component scores for the dataset. Heatmaps **b** and **e** represent the mean component scores for each sex based on treatment groupings. Graphs **c** and **f** illustrate individual data points for each observation are shown along with the mean ± standard error of the mean (SEM). **a)** Chemo-cytokine analysis resulted in three main component groupings. **b) – c)** Male UCMS cecum decreased Component 1 scores in males compared to controls. Female UCMS cecum increased Component 3 scores in males compared to male controls, males treated with male UCMS cecum, and females treated with female UCMS cecum. **d)** Gene expression analysis resulted in two main component groupings. **e) – f)** Overall, males had significantly higher Component 1 scores than females. A significant interaction between sex and treatment revealed that males treated with female UCMS cecum had significantly lower Component 1 scores compared to male controls and male controls had higher Component 1 scores than female controls. *∗ = p < .05*.

Analysis of chemo-cytokine levels within the PFC through PCA resulted in three main component groupings with a significant Bartlett's Test of Sphericity, χ^2^ (105) = 407.521, *p* < .001 (Fig. 3a). Eigenvalues for each component were sequentially 4.93, 3.19, and 1.84, with 32.88, 21.28, and 12.29% of variance explained, respectively. One cytokine, IL-7, was cross-loaded between all three components, while the majority of chemo-cytokines in Component 3 were cross-loaded with Component 2. Furthermore, MIP-2 was cross-loaded between Components 1 and 2.

An ANOVA performed on Component 1 scores identified a significant main effect of treatment, *F* (2,40) = 3.808, *p* = .031, η^2^ = 0.160 (Fig. 3c). Post-hoc analysis revealed that mice treated with male cecum, regardless of recipient sex, had significantly lower scores on Component 1 compared to controls, *p = *.010**,** Cohen's *d* = 0.971, 95% CI [0.241, 1.702]. All chemo-cytokines in this component have loadings >0.5 and are positive in direction.

While no significant main effects or interactions were found for Component 2, *p* > .05, a sex by treatment interaction was found for Component 3, *F* (2,40) = 5.883, *p* = .006, η^2^ = 0.227, with post-hoc tests indicating that males treated with female UCMS cecum had higher scores on this component than male controls, *p = *.005**,** Cohen's *d* = 1.581, 95% CI [0.495, 2.666], males treated with male cecum, *p = *.022**,** Cohen's *d* = 1.276, 95% CI [0.181, 2.371], and females treated with female cecum, *p = *.005**,** Cohen's *d* = 1.555, 95% CI [0.503, 2.607] (Fig. 3c). With loadings >0.5, increases in IL-2 and IL-17a with reductions in LIX (CXCL5) primarily characterize Component 3.

### 3.4 Female UCMS microbiota in male recipients elevates histaminergic and serotonergic signaling while increasing dopamine transporter expression

Analysis of gene expression through PCA resulted in two main component groupings with a significant Bartlett's Test of Sphericity, χ^2^ (28) = 102.043, *p* < .001 (Fig. 3d). Components 1 and 2 had eigenvalues of 2.317 and 2.244, explaining 28.958 and 28.044% of the variance respectively, with a cumulative variance explained of 57.002%. Two genes, Iba-1 and Hrh1, were cross-loaded between components. An ANOVA on Component 1 scores identified a significant main effect of sex, *F* (1,38) = 5.855, *p* = .020, η^2^ = 0.134, with males scoring higher than females, *p = *.004**,** Cohen's *d* = 1.582, 95% CI [0.531, 2.632], and a significant interaction between sex and treatment, *F* (2,38) = 6.930, *p* = .021, η^2^ = 0.183 (Fig. 3f). Post hoc analyses indicated that males treated with female UCMS cecum showed reductions in Component 1 prefrontal cortex chemo-cytokines compared to male controls, *p = *.020**,** Cohen's *d* = 1.127, 95% CI [2.242, 0.192]. Within Component 1, Htrlb and Hrh3 had positive loadings >0.5, while Slc6a3 had a negative loading >0.5; the higher scores among males than females is partially reversed among males receiving female UCMS gut microbiota colonization. No significant main effects or interactions were found for Component 2, *p* > .05.

## 4 Discussion

### 4.1 Overview

Across behavioural, immune, and monoaminergic domains, our findings demonstrate that transfer of UCMS-associated microbiota alters brain and behaviour in a sex-dependent manner. Females consistently showed heightened depressive-like outcomes following microbiota transfer from UCMS mice, whereas males exhibited pronounced neuroimmune and molecular responses in the absence of behavioural change. These divergent patterns highlight distinct pathways through which transfer of stress-exposed microbial communities influence the GBA, shaped by both donor and recipient sex. Together, the results underscore the importance of considering sex as a biological variable when evaluating how microbial perturbations contribute to depression-related phenotypes, how these effects manifest across biological levels, and for whom gut-targeted immunomodulation may be most effective.

### 4.2 Interpretation

Sucrose preference scores on the SPT revealed significantly lower baseline preference in females, and UCMS cecal transfer further reduced sucrose preference in a manner dependent on both donor and recipient sex. Female recipients treated with either male or female UCMS cecum exhibited lower sucrose preference than males receiving the same treatments (Fig. 2a and b). Within female recipients, those receiving cecum from female UCMS donors showed the lowest sucrose preference scores, indicating a heightened susceptibility of the female host–female donor combination to microbiota-associated reductions in reward-related behaviour. These findings align with clinical evidence that inflammatory biomarkers more strongly predict depressive symptoms in women (Jarkas et al., 2024), suggesting that females show greater behavioural sensitivity to microbiota-associated perturbations of reward processing.

Escape-motivation behaviour further supported this interpretation. While males remained behaviourally stable across treatments, females receiving female UCMS cecum showed significantly increased immobility, reduced mobility, and fewer behavioural transitions on the FST (Fig. 2c–f). These effects were more pronounced than those observed in females receiving male UCMS cecum, indicating that the female UCMS microbial profile is particularly potent in reducing behavioural flexibility and promoting passive coping. Females treated with male UCMS cecum also displayed higher mobility than males receiving the same treatment, reinforcing that recipient/host sex also shapes behavioural outcomes. Together, these behavioural data suggest that cecal transfer from UCMS-exposed female donors is associated with increased depressive-like behaviour in female recipients, with females showing the greatest behavioural sensitivity.

Male UCMS cecal transfer was associated with a broad reduction in chemo-cytokine signaling across both sexes, suggesting a dampened pro-inflammatory profile. This pattern was reflected in lower scores on a PCA-derived component composed of GM-CSF, IL-7, IL-12p70, MCP-1, MIP-2, and TNF-α, markers that are often elevated in individuals with MDD (Fig. 3b–c; Capucetti et al., 2020; Dikmen et al., 2020; Nava et al., 2025; Nowak et al., 2019; Yao et al., 2020). In contrast, female UCMS cecal transfer produced a sex-dependent effect, selectively altering immune profiles in male recipients. Males receiving female UCMS cecum exhibited a shift toward a T cell–associated inflammatory signature, characterized by increased IL-2 and IL-17A alongside reduced LIX (CXCL5) (Nadeem et al., 2017; Nava et al., 2025; Zhang et al., 2018). Interestingly, decreases in LIX are linked to increases of corticotropin releasing hormone (CRH) under stress and are associated impairing synapse formation within the hippocampus (Zhang et al., 2018). Since alterations in Component 3 were only seen in males treated with female UCMS cecum, these findings highlight a sex-dependent interaction between microbiota-associated signals and neuroimmune pathways within the gut–brain axis.

Sex-dependent differences in PFC gene expression were observed in association with UCMS-derived microbiota exposure. Baseline comparisons indicated that control males exhibited distinct gene expression profiles relative to control females, which was altered following exposure to female UCMS cecal microbiota (Fig. 3e–f). Genes contributing most strongly to this pattern included positive loadings of *Htr1b* and *Hrh3*, alongside a negative loading of *Slc6a3*. Collectively, this profile is consistent with sex differences in monoaminergic and histaminergic signaling pathways, including serotonergic receptor activity, histamine receptor signaling, and dopamine transporter–related clearance processes (Gontijo et al., 2023; Schlicker and Kathmann, 2016; Qian et al., 2022; Thangam et al., 2018; Tiger et al., 2018). However, males exposed to female UCMS cecal microbiota showed a shift in this multigene expression pattern relative to control males, indicating a microbiota-associated reorganization of gene expression in monoaminergic and histaminergic systems. These findings extend beyond the classical monoamine hypothesis of depression by suggesting that histaminergic signaling, in conjunction with monoaminergic and neuroimmune pathways, may contribute to sex-dependent transcriptional responses to gut-derived signals (Qian et al., 2022). More broadly, the data indicate that the male prefrontal cortex is responsive to microbiota-associated signals under the present experimental conditions, although the functional and behavioural significance of these molecular changes remains to be determined and likely depends on additional interacting factors such as stress exposure or baseline susceptibility.

### 4.3 Limitations

There are several important limitations to consider when interpreting the present findings. The present study did not include microbial sequencing of donor or recipient samples. While sex differences in gut microbiota composition and responses to chronic stress paradigms have been previously reported (Chevalier et al., 2020; Jiang et al., 2025; Kropp et al., 2024), the absence of microbiome profiling here limits direct confirmation of UCMS-associated microbial shifts and engraftment following cecal transfer. Additionally, recipients did not undergo microbial depletion prior to cecal transfer, which has been found to increase efficacy of successful engraftment (Bokoliya et al., 2021). Accordingly, the findings should be interpreted as exploratory with respect to microbiota composition and engraftment. Future studies incorporating both 16S rRNA or metagenomic sequencing in both donors and recipients would help clarify UCMS-induced microbial perturbations and the extent of microbial transfer.

Cecal collection was performed under aerobic conditions and without cryoprotectants. Given that many gut microbes are obligate anaerobes, oxygen exposure may reduce viability of specific taxa and alter community composition relative to the in vivo state (Bokoliya et al., 2021). Although samples were immediately suspended in PBS and stored at −80 °C, the absence of cryoprotective agents such as glycerol may further affect long-term microbial preservation, with evidence supporting their benefit under frozen storage conditions (Starr et al., 2014). Also, saline gavage was used as a procedural control for oral administration; however, this does not control for effects attributable specifically to microbial exposure. Additionally, collected cecum was pooled by sex, with cecal recipients receiving the full range of pooled donor microbiome inoculum, limiting the effects of donor cecal profile variation. Inclusion of an additional control cecal donor group along with constraining the pool of donors per cecum recipient would strengthen causal inference regarding UCMS-derived microbiota effects.

Behavioural testing began 24 h after completion of cecal transfer, which may have influenced the magnitude or detectability of microbiota-associated behavioural effects. Although behavioural alterations were detected, the changes observed early post-transfer may not be representative of a stabilized microbial profile (Bokoliya et al., 2021). Future studies incorporating later or repeated behavioural assessments would elucidate short-lived or dynamic post-transfer effects.

There was also a modest age difference between donor and recipient animals at the time of transfer. Although the gut microbiome is generally considered relatively stable across early to mid-adulthood (Jiao et al., 2025), age-related variability cannot be fully excluded. Future work using age-matched donor and recipient cohorts would help address this potential confound. Finally, estrous cycle stage was not tracked in female recipient animals. While microbial composition has not consistently been shown to fluctuate across the estrous cycle (Pestana and Graham, 2024; Wallace et al., 2018), hormonal state may influence behavioural outcomes. Future studies incorporating estrous cycle monitoring would allow more precise interpretation of sex-dependent behavioural effects.

### 4.4 Conclusion

Overall, our findings support prior work showing that transfer of gut microbial profiles associated with MDD or chronic stress is associated with development of a depression relevant phenotypes in otherwise unmanipulated mice, while extending this literature by demonstrating that sex critically shapes donor–recipient outcomes (Chevalier et al., 2020; He et al., 2024; Hinks et al., 2023; Kropp et al., 2024; Li et al., 2019; Marin et al., 2017; Zhou et al., 2023). Generally, this provides evidence suggesting that interactions of sex-specific gut microbiota contribute to behavioural and neuroimmune phenotypes associated with depression and highlight the need to identify the taxa and pathways responsible for these effects. Transfer of UCMS-exposed microbiota produced sex-dependent behavioural effects, with female-derived microbiota more readily increasing anhedonia and reducing escape-motivated behaviour in female recipients. Such findings suggest that gut-targeted interventions may be particularly beneficial for females, in whom stress-altered microbiota was significantly associated with depressive-like behavioural phenotypes. In males, alterations in T-cell signaling (i.e., IL-2, IL-17a) may reflect stress-related immune activation, functioning as biomarkers of vulnerability or, alternatively, indicating compensatory protective mechanisms that warrant further investigation. Additionally, males may require additional biological or environmental factors - such as heightened inflammatory load, chronic stress exposure, or microbial perturbation-for immune activation to manifest behaviourally. Nonetheless, this pattern highlights a sex-dependent dissociation between immune signaling and behaviour, reinforcing the idea that inflammation contributes to depressive-like phenotypes only when aligned with sex-specific host vulnerability. This work underscores the importance of incorporating sex at multiple biological levels - microbial, immune, and neural - when evaluating immune contributions to depression. Rather than directly driving depressive-like behaviour, inflammation in this model may represent an early or permissive state that precedes behavioural changes and interacts with sex-specific host biology and gut microbial composition to shape later outcomes.

## CRediT authorship contribution statement

**Meagan E. Hinks:** Conceptualization, Data curation, Formal analysis, Investigation, Methodology, Supervision, Visualization, Writing – original draft, Writing – review & editing. **Alexandre S. Maekawa:** Data curation, Investigation, Methodology, Writing – original draft, Writing – review & editing. **Mark D. Corrigan:** Data curation, Methodology. **S.M. Nageeb Hasan:** Data curation, Methodology, Writing – review & editing. **Derek Wan-Yan-Chan:** Data curation, Investigation, Methodology, Writing – review & editing. **Tanya Nadine Burry:** Data curation, Investigation, Methodology, Writing – review & editing. **Stephanie Salia:** Data curation, Formal analysis, Investigation, Methodology, Writing – review & editing. **Francine F. Burke:** Data curation, Investigation, Methodology, Writing – review & editing. **Francis R. Bambico:** Conceptualization, Formal analysis, Methodology, Supervision, Writing – review & editing. **Ashlyn Swift-Gallant:** Conceptualization, Funding acquisition, Investigation, Methodology, Project administration, Supervision, Writing – original draft, Writing – review & editing.

## Funding

Research was supported by a Discovery Grant from the Natural Sciences and Engineering Council of Canada (NSERC; RGPIN 2019-04999) and Canadian Institutes of Health Research (CIHR; 495842) Project Grant to AS-G.

## Declaration of competing interest

The authors have no conflicts to declare.

## Data Availability

Data will be made available on request.
