# Supplementary material for: Both donor and recipient sex determine behavioral and neuroimmune outcomes in a gut microbiota transfer experiment from the unpredictable chronic mild stress model of depression
Source: Brain Behav Immun Health. 2026 Apr 27;54:101248. doi: 10.1016/j.bbih.2026.101248 (PMC13153600; doi:10.1016/j.bbih.2026.101248)
Supplement: Multimedia component 1 [file mmc1.docx]

**Method**

**Animals**

Mice of the Balb/C strain used for both the cecum donor and recipient groups were obtained from Charles River Laboratories (St. Constant, QC, CA). Mice were housed on a Digitally Ventilated Cage (DVC) rack (Tecniplast, West Chester, PA, USA) set at 21°C and 33-46% humidity on a 12-hour light/dark cycle (lights on 7:00am). All mice were singly housed upon arrival to the facility to control for cross-mouse coprophagy and cage effects on the microbiome in the recipients (Russell et al., 2022). Standard pellet chow (Harlan Teklad 2018 Rodent Diet, Indianapolis, IN, USA) and water were provided *ad libitum*. Fresh water was supplied weekly and cage changes were performed every two weeks. Ethics approval of this study was acquired through the Institutional Animal Care Committee at Memorial University of Newfoundland under compliance with Canadian Council of Animal Care (CCAC) guidelines.

***Cecum Donor Mice***

Cecum donor mice (n = 6-8 per sex) underwent a 6-week UCMS paradigm beginning at post-natal day (PND) 60. Stressors consisted of a variety of physical (i.e., cage tilt), psychological (i.e., predator scent), and circadian (i.e., stroboscope overnight) stressors, all inescapable but non-debilitating in nature and were implemented three times a day, five days a week modified from Bambico et al., 2009. To determine whether UCMS was sufficient in inducing a depressive-like model, the Sucrose Preference Test was performed weekly. UCMS was deemed successful when the sucrose preference scores of the UCMS group were significantly lower than those of a non-UCMS control group. To further ensure the efficacy of the UCMS paradigm, an additional week was implemented following validation of UCMS through sucrose preference. Cecum collection occurred one day post-UCMS completion during culling through excision of the cecal sac from the colon via a medial incision from the sternum to pelvis and microbial contents were extracted from the sac into a 1.7mL centrifuge tube. Donor mice were overdosed through intraperitoneal injection of Avertin (40mg/kg) into the lower right quadrant of the abdomen to avoid injection into the cecum and then underwent rapid decapitation. Samples were pooled by sex 2-3 mice per tube and diluted 1:1 by volume with 0.1M sterile phosphate buffered saline (PBS) for stabilization upon collection. Samples were immediately placed on dry ice for temporary storage prior to being stored at -80 long-term until cecal transfer. Prior to recipient transfer, samples were thawed and further diluted to a 1:10 cecum to saline ratio.


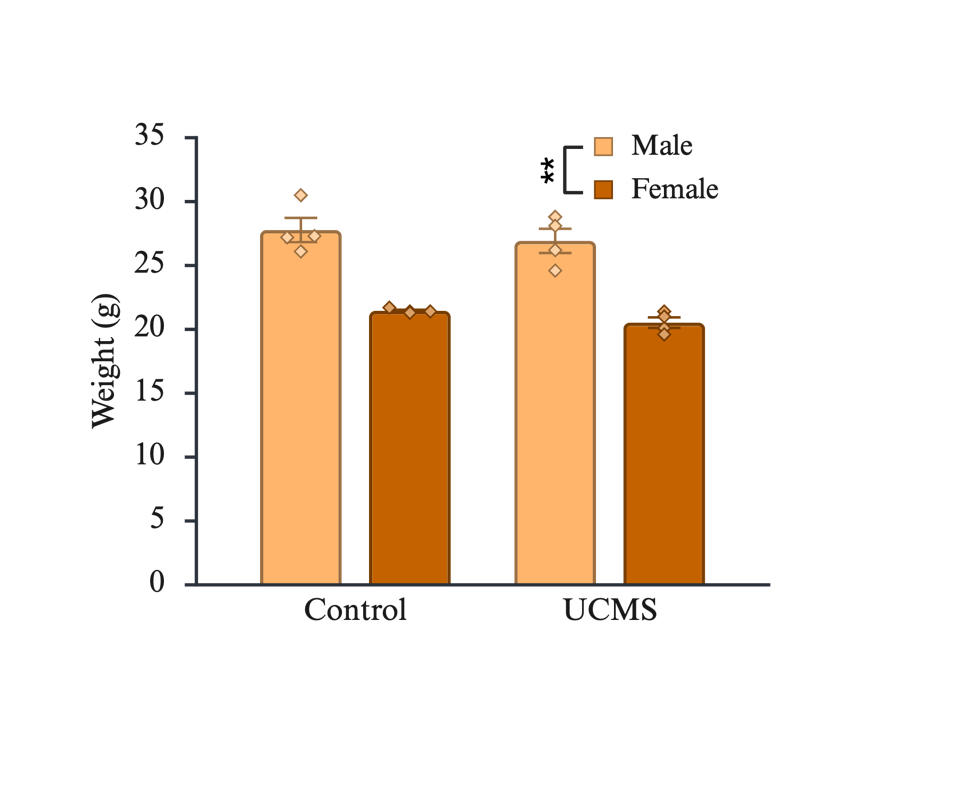
***Cecum Recipient Mice***

**Figure 1.** Weights for a subset of UCMS cecum donor mice compared to a non-UCMS control group at the time of dissection. Note*.* Individual data points for each observation are shown along with the mean ± standard error of the mean (SEM). Male mice weighed significantly more than female mice F (1,12) = 1.593 *p* < .001, η^2^ = 0.117. ** = p < 0.05, ** = p < .001*

To determine whether colonization with microbial contents from a UCMS depression model is sufficient to induce downstream GBA and behavioural alterations in recipient mice, sixty Balb/c mice were administered 0.2ml of cecal solution or saline via oral gavage using a 20-gauge ball-tipped stainless steel gavage needle every other day for 12 days (6 treatments total; as previously described, Hinks et al., 2023). Treatment began at PND 60-65, and an equal number of males and females were assigned to the following treatments: male cecum, female cecum, or saline (control; n = 10 males and females/group). In the 3 days following the last cecal transfer, behaviour testing took place, as described below, and on the fourth day post-treatment, mice were culled for brain collection. Trunk blood was collected and brains were extracted from the skull and flash frozen using 2-methyl butane prior to being stored at -80 until further processing.

**Behavioural Testing**

Behavioural testing commenced 24 hours after the final gavage session to assess for the presence of anxiety- and/or depressive-like behaviours. Mice underwent a battery of four standard behavioural tests: Sucrose Preference Test (SPT), Splash Test (ST), Tail Suspension Test (TST), and Forced Swim Test (FST). The testing battery was administered across three days: day one involved SPT habituation, day two comprised SPT testing, and day three concluded with the ST, TST, and FST.

***Sucrose Preference Test***

The SPT is a standard behavioural test used to assess anhedonia-like behaviour in preclinical rodent models (Papp et al., 1991). Anhedonia is characterized by a loss of motivation to participate in, and a lack of enjoyment of, previously pleasurable activities, and is a core symptom of MDD (American Psychiatric Association [APA], 2013; Treadway & Zald, 2011). In rodents, this is modelled through a reduction of preference for a sucrose solution over water (Wulff et al., 2023). Day one consists of habiting the mice to the presence of two 1.5-ounce cups within the cage. During this phase, each cup is filled with drinking water and secured to the base of the home cage with masking tape for 18 hours. On day two of testing, the cups are refilled, one with a 4% sucrose solution and the other with water; prior to placement in the cage, both cups are are weighed. Side placement of the sucrose containing cup was counterbalanced between sexes and treatment groups. After the 18-hour testing period, the final weights of the cups were recorded and deducted from the initial weights to calculate overall consumption. Sucrose preference scores were then calculated by dividing the weight of sucrose consumed by the total weight of liquid consumed, with a low preference score defined as less than 70% (Wulff et al., 2023). Data from four females, two saline and two female cecum treated, were excluded due to loss of sample during the testing phase.

***Splash Test***

The ST uses grooming behaviours in the presence of a soiled coat as an indicator of motivation and self-care behaviours, behaviours often reduced in MDD diagnosis (APA, 2013; Yalcin et al., 2005, 2008). For the ST mice were placed in an empty cage and their dorsal coat was saturated with 0.2mL of a syringed 20% sucrose solution. Grooming behaviours were then video recorded for five minutes. Behavioural scoring was performed manually using the BORIS (Version 7.13.9 MacOS) software and grooming was defined into two categories: general and back grooming (Friard & Gamba, 2016). Back grooming was defined as the mouse grooming/licking their dorsal side, from the base of their neck to the top of their hind- and forelimbs, while general grooming consisted of all other areas, including the tail, face and hands.

***Tail Suspension Test and Forced Swim Test***

The TST and FST analyze motivation to escape distressful situations through measures of mobility as a proxy for despair (Fitzgerald et al., 2019; Lad et al., 2007). In the TST mice were suspended from a wire cage lid by their tail. The tail is secured with masking tape 0.5 cm from the tip and hung from the lid, ensuring that they cannot touch the base or walls of the cage. For the FST, transparent glass cylinders (12.0cm in diameter x 30cm height) were filled with water (temperature 25 ◦C ± 0.5 ◦C) to a level that ensured they could not reach the top or bottom of the cylinder. For both the TST and FST, mice are suspended and video recorded for five minutes during which they are monitored (i.e., prevent tail climbing, falling to the base of the cage, submersion in the water). Video recordings were coded for mobility measures using the automated Ethovision XT14 software (Noldus et al., 2001). Mobility was scored for duration and frequency bouts of immobility and high mobility.

**Brain Tissue Processing**

On dry ice, the PFC was sectioned from each brain using a coronal slice at the level of the medial cerebral artery using a sterilized razor blade. Each PFC was then subdivided medially at the longitudinal fissure and the olfactory bulbs removed and stored at -80 in 1.7 mL microcentrifuge tubes until further processing. Each half of the PFC was homogenized separately prior to being used for either chemo-cytokine or gene expression analysis.

***Chemo-Cytokine Analysis***

Half of the PFC samples were analyzed in duplicate using the Mouse High Sensitivity T-Cell 18-Plex Discovery Assay® Array (MDHSTC18, Eve Technologies, Calgary, AB, Canada), detecting a range of pro-inflammatory cytokines and chemokines. Sensitivity range for the samples was 0.06-9.06 pg/mL. For homogenization, samples were transferred from the microcentrifuge tubes to bead mill tubes (**Cat. No.** 15-340-154). Tissue lysates were prepared by calculating the required volume of digestion buffer at 10 µL per gram of sample. Pierce RIPA Buffer (Cat. No. 89900) was supplemented with Halt Protease and Phosphatase Inhibitor (100X; Cat. No. 1861281), prepared by adding 10 µL of inhibitor to every 1000 µL of RIPA and kept on ice throughout the procedure. Digestion buffer was added to each sample tube, and tissues were homogenized using a bead-mill grinder (speed five for 15 seconds; Cat. No. 12-1413-62) until no visible tissue fragments remained. Homogenates were pipetted from the bead mill tubes into microcentrifuge tubes and centrifuged at 15,000 RCF for 10 min at 4 °C, after which the clarified supernatant was carefully transferred to fresh tubes without disturbing the pellet. Protein extracts were stored at −20 °C until being sent off for analysis.

***Gene Expression Analysis***

The other half of the PFC was analyzed using quantitative polymerase chain reaction (qPCR) to assess expression of a variety of genes linked to MDD. Tissue homogenization and total RNA purification of these samples was performed using the PureLink™ RNA Mini Kit (Cat. No. 12183025). Brain tissue was homogenized using the bead mill tubes and bead-mill grinder method (Grinstein et al., 2018). Tissue was placed in the bead mill tubes along with 600 µL of Lysis Buffer supplemented with 2-mercaptoethanol (1 mL: 10 µL) and homogenized at speed five for 15 seconds. Following homogenization, RNA was extracted according to the manufacturer’s protocol for frozen tissue, starting at Step Two (Pub. No. MAN0019346). RNA quality was verified using the Thermo Scientific™ NanoDrop™ One Spectrophotometer (Cat. No. 13-400-518). Samples with RNA concentrations greater than 150 ng/µL and purity ratios close to 2.0, 260/280 and 260/230, were used for further qPCR analysis.

Prior to generation of cDNA, each sample was further purified using ezDNase™ Enzyme (Cat. No.  11766051; Pub. No. MAN0015899) for degradation of genomic DNA and synthetization of cDNA was performed using the SuperScript™ IV Reverse Transcriptase kit (Cat. No. 18090050; Pub. No. MAN0013443). qPCR reactions were performed using the CFX Connect Real-Time PCR Detection System (Bio-Rad, Cat. No. 185-5200). cDNA was diluted to a final dilution of 1:100 with deionized water. For each reaction, 10 µL of TaqMan™ Universal PCR Master Mix (Cat. No. 4304437) and 1 µL of the corresponding TaqMan™ Gene Expression Assay tagged with FAM were combined with 9 µL of cDNA (total vol. 20 μL). Each reaction was performed in triplicate, with GAPDH labelled with VIC as an endogenous control. List of primers can be found in Table 1.

**Table 1**

List of genes and their corresponding primers.

| **Gene name** | **Gene symbol** | **TaqMan Assay ID (Catalog)** |
| --- | --- | --- |
| Allograft inflammatory factor 1 | Iba1, Aif1 | Mm00479862_g1 |
| Brain-derived neurotrophic factor | Bdnf | Mm04230607_s1 |
| Cluster of differentiation 68 | Cd68 | Mm03047343_m1 |
| Dopamine transporter (solute carrier family 6 member 3) | Slc6a3 | Mm00438388_m1 |
| 5-Hydroxytryptamine (serotonin) receptor 1A | Htr1a | Mm00434106_s1 |
| 5-Hydroxytryptamine (serotonin) receptor 1B | Htr1b | Mm00439377_s1 |
| Histamine receptor H1 | Hrh1 | Mm00434002_s1 |
| Histamine receptor H3 | Hrh3 | Mm00446706_m1 |
| Glyceraldehyde-3-phosphate dehydrogenase (housekeeping control) | Gapdh | Mm99999915_g1 |

**Statistics**

All statistical analyses were performed using Jamovi (Version 2.6.45; The Jamovi project, 2025). Differences in behavioural measures were assessed using between-subjects analysis of variance (ANOVA) to compare groups based on sex, treatment, and sex by treatment interactions. For analyses of chemo-cytokine and gene expression, a Principal Component Analysis (PCA) using an Oblimin rotation (δ = 0) was applied. Parallel analysis was performed on each measure to determine component retention. Bartlett’s Test of Sphericity was performed for each PCA to confirm correlation within each set of factors. For chemo-cytokines expression, raw values were used, while gene expression levels were determined by calculating the difference in expression between the target gene and GAPDH. Component scores were saved, and an ANOVA was run on each to evaluate whether mice differed on their component scores based on sex and/or treatment. Significant effects were followed up by Fishers Least Significant Difference (LSD) using planned comparisons of treatments within sex and treatments across sex, with critical alpha set at *p* < .05. Post hoc p‑values were corrected for multiple comparisons using the Benjamini–Hochberg false discovery rate (FDR) procedure (*q* < 0.15).

**References**

American Psychiatric Association [APA]. (2013). *Diagnostic and statistical manual of mental disorders* (5^th^ ed.). <https://doi.org/10.1176/appi.books.9780890425596>

Bambico, F. R., Nguyen, N.T., & Gobbi, G. (2009). Decline in serotonergic firing activity and desensitization of 5-HT1A autoreceptors after chronic unpredictable stress. *European Neuropsychopharmacology, 19*(3), 215–228. <https://doi.org/10.1016/j.euroneuro.2008.11.005>

Fitzgerald, P. J., Yen, J. Y., & Watson, B. O. (2019). Stress-sensitive antidepressant-like effects of ketamine in the mouse forced swim test. *PLOS ONE*, *14*(4), e0215554. https://doi.org/10.1371/journal.pone.0215554

Friard, O., & Gamba, M. (2016). BORIS : a free, versatile open‐source event‐logging software for video/audio coding and live observations. *Methods in Ecology and Evolution*, *7*(11), 1325–1330. <https://doi.org/10.1111/2041-210X.12584>

Lad, H. v., Liu, L., Payá-Cano, J. L., Fernandes, C., & Schalkwyk, L. C. (2007). Quantitative traits for the tail suspension test: automation, optimization, and BXD RI mapping. *Mammalian Genome*, *18*(6–7), 482–491. <https://doi.org/10.1007/s00335-007-9029-1>

Noldus, L. P. J. J., Spink, A. J., & Tegelenbosch, R. A. J. (2001). EthoVision: A versatile video tracking system for automation of behavioral experiments. *Behavior Research Methods, Instruments, & Computers, 33*(3), 398–414. <https://doi.org/10.3758/BF03195394>

Papp, M., Willner, P., Mus, R. (1991). An animal model of anhedonia: attenuation of sucrose consumption and place preference conditioning by chronic unpredictable mild stress. *Psychopharmacology, 104*(2), 255–259. <https://doi.org/10.1007/BF02244188>

Russell, A., Copio, J. N., Shi, Y., Kang, S., Franklin, C. L., & Ericsson, A. C. (2022). Reduced housing density improves statistical power of murine gut microbiota studies. *Cell Reports*, *39*(6), 110783. <https://doi.org/10.1016/j.celrep.2022.110783>

The Jamovi project (2025). *Jamovi* (Version 2.6.45) [Computer Software]. Retrieved from https://www.jamovi.org.

Treadway, M. T., & Zald, D. H. (2011). Reconsidering anhedonia in depression: Lessons from translational neuroscience. *Neuroscience & Biobehavioral Reviews, 35*(3), 537–555. <https://doi.org/10.1016/j.neubiorev.2010.06.006>

Wulff, A. B., Cooper, P., Kodjo, E., Abel, E., & Thompson, S. M. (2023). How Sucrose Preference Is Gained and Lost: An In-Depth Analysis of Drinking Behavior during the Sucrose Preference Test in Mice. *eNeuro, 10(*9). <https://doi.org/10.1523/ENEURO.0195-23.2023>

Yalcin, I., Aksu, F., & Belzung, C. (2005). Effects of desipramine and tramadol in a chronic mild stress model in mice are altered by yohimbine but not by pindolol. *European Journal of Pharmacology*, *514*(2–3), 165–174. <https://doi>.org/10.1016/j.ejphar.2005.03.029

Yalcin, I., Belzung, C., & Surget, A. (2008). Mouse strain differences in the unpredictable chronic mild stress: a four-antidepressant survey. *Behavioural Brain Research, 193*(1), 140–143. <https://doi.org/10.1016/j.bbr.2008.04.021>
